# Supplementary material for: Role of leukotriene B4 (LTB4)-LTB4 receptor 1 signaling in post-incisional nociceptive sensitization and local inflammation in mice
Source: PLoS One. 2022 Oct 20;17(10):e0276135. doi: 10.1371/journal.pone.0276135 (PMC9584502; doi:10.1371/journal.pone.0276135)
Supplement: Attachment — Submitted filename: Responses to the Reviewers .docx [file pone.0276135.s001.docx]

We appreciate all reviewers for reviewing our manuscript and giving useful comments to improve our manuscript.

Reviewer #1: The manuscript by Asahara investigates the contribution of BLT1 in the development of mechanical pain hypersensitivity produced by incision and the related inflammation. The authors show that Blt1 knock-out animals express reduced pain hypersensitivity after incision compared to wild-type animals. They also report that incision produces, in a time-dependent manner, an increase in LTB4 in the plantar tissue. The genetic deletion of Blt1 reduces the infiltration of monocytes and the production of IL-1beta and TNFalpha in the plantar tissue.

Overall, the topic addressing the mechanisms responsible for surgical pain is interesting. The results are clearly presented and the manuscript easy to read. An important issue here is the mechanisms of action of BLT1 that is not clearly investigated making the reading of the manuscript quite confusing. Indeed, the authors suggest a local mechanisms involving BLT1 expressed in immune cells promoting the recruitment of monocytes. However, BLT-1 is also express in sensory neurons of the DRG and the spinal cord, as reported by the authors. Hence, the experiments and the data presented in the manuscript do not allow to conclude on how BLT1 participates in the development of incision-induced pain hypersensitivity. Extensive revision is needed to address such an important issue. For instance, additional experiments evaluating the effects of intraplantar LTB4 injection on pain sensitivity, monocytes infiltration and cytokines production could be performed.

We evaluated the effects of LTB_4_ injection on pain sensitivity (Fig. 5), and we observed transient pain responses. We speculated that peripherally-increased LTB_4_ induces pain-related responses via both the BLT1 pathway, expressed in inflammatory monocytes, and the TRPV1 pathway, expressed in the afferent sensory nerves of the skin. However, it was unclear whether LTB_4_ directly acted on TRPV1 because LTB_4_ disappeared rapidly. We believe that the direct effect of LTB_4_ on the peripheral nerve endings in the skin will not be long-lasting. Based on our results, we propose that LTB_4_-induced inflammation, mainly the recruitment of inflammatory monocytes, is more strongly involved than TRPV1 activation of peripheral nerves by LTB_4_ in the development of incision-induced hypersensitivity.

The additional comments should be considered by the authors.

- Additional experiments with the use of a non-reflexive test should be conducted. The sole description of pain-related behaviors via the use of reflexive test is insufficient to conclude on alteration of pain behaviors. For instance, pain score, place preference paradigm, catwalk etc…are methods used to assess pain-related behaviors without the use of a reflexive response.

Unfortunately, because of the shortage of mice, we were unable to perform the non-reflexive test to compare the behaviors of BLT1KO and BLT1WT mice. As suggested by the reviewers, we have evaluated the effects of intraplantar LTB_4_ injection on pain sensitivity using the guarding pain score, which enabled us to assess spontaneous pain-related behaviors. (Fig. 5)

- The effects of Blt1 deletion seems moderate and transient on the mechanical pain hypersensitivity. One may question on the importance of Blt1 in incision-induced pain-related behaviors, especially from a clinical point of view. The authors should comment.

Although NSAIDs are standard analgesics administered for postoperative pain, their use can present challenges because of side effects, such as aspirin-induced asthma and gastrointestinal damage. A BLT1 antagonist may be effective as an anti-inflammatory analgesic secondary to NSAIDs, with fewer side effects during the early phase of postoperative pain. Additionally, treatments targeting LTB_4_-BLT1 signaling in inflammatory monocytes (the Ly6C^high^ subset) may be useful for decreasing inflammation to inhibit pain. (L346-353)

- To exclude potential compensatory mechanisms in Blt1 Knock-out mice, pharmacological experiments targetting BLT1 should be considered by the authors. For instance, the effects of intraplantar injection of the BLT1 antagonist ONO-4057 could be evaluated in the incisional pain model.

We could not obtain a sufficient amount of the BLT1 antagonist to perform the experiments because ONO-4057 is not commercially available. However, we hypothesize that intraplantar administration of a BLT1 antagonist would attenuate incisional pain because painful behavior was observed after intraplantar injection of LTB_4_. Moreover, local BLT1 antagonist administration may have an inhibitory effect on inflammatory cell infiltration and cytokine increase.

- Have the experiments been performed in a blinded manner ? This should be indicated in the manuscript. If not, the authors should justify.

The experiments were performed in a blinded manner. We have described this in the text. (L130 and142)

- It is admitted that the use of the up and down method for the the von frey test allows to evaluate not hyperalgesia but allodynia. Actually, for the description of the results the terms "Mechanical hyperalgesia" should be changed by "mechanical pain hypersensitivity" as the reflexive test does not allow to truly assess hyperalgesia/allodynia in animals.

We changed the term “Mechanical hyperalgesia” by “Mechanical pain hypersensitivity”.

- Plantar tissue has been collected to perform biochemical and cellular analysis. However, it is unclear what represents this tissue. Does this include muscle and skin or just the plantar muscle? The authors should comment.

Description of “Plantar tissue” was added to the method section, as “Plantar tissue, including skin and underlying muscle.”

Reviewer #2: Miho Asahara and colleagues evaluated LTB4/BLT1 in post-incisional pain.

Major points

The role of BLT1 was evident at days 2, 3 and 4 post-incision (Figure 1). Why LTB4 production was quantitated at 3h and days 1 and 7? Using the same tool, the authors can ascertain the LTB4 production at time points that were observed as important since BLT1 deficiency caused a reduction of mechanical allodynia at days 2, 3 and 4 post-incision (Figure 1). It is possible that the role of LTB4/BLT1 has consequences that goes beyond the time point of LTB4 production. However, the lack of data at those days do not allow reaching a conclusion. Overall, there seems to be an arbitrary selection of time points of analyzes that prevent putting all data in line to evaluate them. Figure 1 checked many time points, figures 2 and 4 verified 3h, 1 and 7 days, and figure 3 verified 3h, 1, 3 and 7 days.

Why LTB4 production was evident at 3h and then significant alteration of pain was observed only at 2, 3 and 4 days later? Note that this is a difference of many days. It would be interesting to check if injection of LTB4 produces a similar profile.

Additional experiments on the quantification of LTB_4_ concentration revealed that LTB_4_ was detected on days 1 and 3. As suggested by several reviewers, we have evaluated the effects of LTB_4_ injection on pain sensitivity, and we observed transient pain responses. We speculate that peripherally-increased LTB_4_ induces pain-related responses via both the BLT1 pathway, expressed in inflammatory monocytes, and/or the TRPV1 pathway, expressed in the afferent sensory nerves of the skin.

Quite interestingly, inflammatory monocytes were increased at days 1 and 3, and their recruitment was inhibited by BLT1 deficiency. These results suggest a parallel between pain and inflammatory monocyte recruitment. However, the data do not demonstrate whether at all time points with difference in phenotype there is also difference in LTB4 production. This is important to be done and further supports the question above.

We quantified LTB_4_ concentration in the plantar tissue on days 1 and 3 after the incision, the results of which are provided in the new Fig. 2. LTB_4_ concentration peaked at 3 h, and a moderate level of LTB_4_ was detected not only on day 1 but also on day 3, which indicated that the LTB_4_ increase affected Ly6C^high^ monocyte recruitment and strengthened inflammation on days 1 and 3.

In regard to the reasons why the pain response in BLT1KO mice was not suppressed on day 1, we speculate that the inflammatory reaction was relatively strong on day 1, and stronger monocyte suppression was necessary for pain inhibition during this period.

Furthermore, which is the cellular target of LTB4? Do Ly6Chigh inflammatory monocytes express BLT1? Is it a direct effect (or lack of effect in the case of BLT1 deficient mouse) of LTB4 in BLT1 expressed by Ly6Chigh inflammatory monocytes? Are Ly6Chigh inflammatory monocytes producing the cytokines?

In general, BLT1 is highly expressed in neutrophils, and LTB_4_-BLT1 signaling in neutrophils is the primary initial inflammation response. Consequently, a cascade of the accumulation of neutrophils and other inflammatory cells is induced. In our previous report (Ref. 52), the expression of BLT1 was confirmed in CCR2-high inflammatory monocytes, which were identified as Ly6C^high^ inflammatory monocytes. We propose that Ly6C^high^ inflammatory monocytes are a target of LTB_4_ and that LTB_4_-BLT1 signaling in inflammatory monocytes plays an important role in the progression of inflammation.
Furthermore, according to the reference mentioned below (doi: 10.1186/s12990-015-0010-9), MPO-rich Ly6C^high^ monocytes promote inflammation and tissue destruction via the release of proteases and inflammatory cytokines, such as TNF-α, in various inflammatory settings.

The discrepancy between the present finding and the previous finding (DOI: 10.1073/pnas.1501372112) should be better explored with experiments. These papers are suggesting different cellular populations are involved in incisional pain. What are Ly6Chigh inflammatory monocytes doing in incisional pain and how LTB4 can affect their function? What is the explanation for the difference at day 1? At this time point there is no significant analgesia by BLT1 deficiency, but there is reduction of Ly6Chigh inflammatory monocytes and IL-1b and TNFa production. However, Ly6Chigh inflammatory monocytes are high at day 3 and we do not know the cytokine profile.

Previous reports from other group (DOI: 10.1073/pnas.1501372112) have shown that monocytes (nonneutrophil myeloid cells) are more involved than neutrophils in postoperative pain. In our study, we showed that inhibition of monocytes by suppressing BLT1 signaling also contributes to pain control. BLT1-deficiency resulted in the suppression of monocyte infiltration on days 1 and day 3, and may also be involved in the suppression of cytokines on day 1.

Regarding the reason why the pain response in BLT1KO was not suppressed on day 1, we speculate that inflammatory reaction was relatively strong on day 1; thus, the stronger monocyte suppression may be necessary for pain inhibition.

The data of cytokine production at day 3 was added to the new Fig 4.

The current data do not allow to conclude if the phenotype of BLT1 deficiency is a result of direct function of LTB4 in neurons or non-neuronal cells or if it depends on indirect effect of tissue resident cells, which produce other molecules that are the actual actors to produce pain and inflammatory cell recruitment or both. LTB4 can exert neuronal and non-neuronal effects that will lead to pain. The neuronal mechanism (DOI: 10.1016/j.molbrainres.2005.02.029) was clearly not investigated in the present study and the authors used a model of pain.

We focused on the role of inflammatory cells and LTB_4_-BLT1 signaling in an incisional pain model. It remains unclear how peripherally-produced LTB_4_ acts on the DRG; however, it is possible that LTB_4_ activates BLT1 expressed in the small TRPV1-positive neurons of the DRG and mediates pain sensitization. A description of this has been added to the discussion section. (L369-373, L387-388)

What is the level of LTB4 in the spinal cord? These authors have shown the increase of pCREB in the spinal cord in the formalin test is BLT1 dependent. What happens in the incisional pain model?

In this study, the effect of LTB_4_-BLT1 signaling on central sensitization, such as an increase of pCREB in the dorsal horn of the spinal cord, was not confirmed. We focused on the peripheral mechanisms, determined which types of inflammatory cells are involved, and investigated whether LTB_4_ is produced at the site of injury and whether inflammatory cell infiltration is affected by the deficiency of the LTB_4_ receptor BLT1. LTB_4_ was not detected in the spinal cord on day 3 of the incisional pain model (data not shown). BLT1 expressed in the small neurons of the DRG may have impacted the modulation of incisional pain.

In a prior study, the authors used the MPO assay (doi: 10.1186/s12990-015-0010-9). To give a more comparative view of the results and align them with the literature, it is important to have MPO data and show which cells are producing MPO in the present study. Do these cells express the BLT1 receptors?

According to the single-cell RNASeq data (<http://bis.zju.edu.cn/MCA/search2.html>), cluster 17 with high expression of BLT1 is a neutrophil with high MPO and also expresses Ly6C1 and Ly6C2. In addition, cluster 5 macrophages express Ly6C2, BLT1, and MPO. These data indicated that neutrophils and monocytes/macrophages express both BLT1 and MPO, and release MPO via LTB_4_-BLT1 signaling. Although it was difficult in our study to determine whether the source of MPO is neutrophils or monocytes, MPO release may contribute to inflammation and pain enhancement.

Do inflammatory monocytes (Ly6Chigh) produce MPO in the present experimental condition? Please, see (doi: 10.1172/JCI42304).

Thank you for the information about inflammatory monocytes (Ly6C^high^) and MPO production. The study reported that inflammatory monocytes (Ly6C^high^) express a 10-fold higher level of MPO than non-classical monocytes (Ly6C^low^). We have described this in the discussion section. (L360-368)

Figure 2. The number of samples ranged from 4 to 7. It is a huge variation in the number of samples. Why?
A huge variation also occurred in Figure 4 with n varying from 5 to 9. Why?
There is no mention on repetition of experiments.
There is no mention about data normality and homogeneity.

We quantified LTB_4_ concentration in the paw at the missing time points, and the sample ranges have been corrected. Individual data are shown in Fig. 2, which illustrates the variation of the data of each sample.

We apologize for the incorrect sample size provided in Fig. 4. We have corrected the sample size, and individual data are shown in the figure to illustrate the variation of the data of each sample.

Figures 1 and 2 can be combined.

Reviewer #3: In this study, the authors have shown that plantar incision-induced mechanical hypersensitivity, accumulation of inflammatory monocytes and the production of IL-1β and TNF-α in the incised area were significantly reduced in BLT1-knockout mice. Although the present results can be partly expected from previous findings, this study provides further insights into the roles of LTB4-BLT1 signaling in postoperative incisional pain. However, there are some points that should be addressed by the authors, as described below.

1) To indicate that this study is basic research using experimental animals, the animal species should be included in the title.

The animal species used in our study have been added to the title.

2) The basal 50% paw withdrawal threshold in C57BL/6 mice is usually less than 1 g. Have you made any mistakes in the calculations in up-down method of von Frey filament test? Provide the detailed methods in the test and the calculated method.

The details of the methods of assessment and calculation of the mechanical responses are described in the method section. (L104-121)

3) Significant reduction of mechanical hypersensitivity in BLT1KO mice was still not observed at 2h and 1d, but peaked between 2d and 4d after the plantar incision. By contrast, the accumulation of inflammatory monocytes and the production of IL-1β and TNF-α were already reduced at 1 day after the plantar incision. How do the authors explain this time gap? The authors should discuss this point in Discussion section.

We quantified LTB_4_ concentration in the plantar tissue on days 1 and 3 after the incision, and the results have been added to the new Fig. 2. LTB_4_ concentration peaked at 3 h and was still detectable on day 3, which indicated that a persistent LTB_4_ increase affects Ly6C^high^ monocyte recruitment and strengthens inflammation on days 1 and 3. BLT1-deficiency suppressed monocyte infiltration on days 1 and 3, and may also be involved in the suppression of cytokines on day 1.

Regarding the reasons why the pain response in KO mice was not suppressed on day 1, we speculate that the inflammatory reaction was relatively strong on day 1 and, thus, stronger monocyte suppression may have been necessary for pain inhibition during this period.
