# Supplementary material for: Role of leukotriene B4 (LTB4)-LTB4 receptor 1 signaling in post-incisional nociceptive sensitization and local inflammation in mice
Source: PLoS One. 2022 Oct 20;17(10):e0276135. doi: 10.1371/journal.pone.0276135 (PMC9584502; doi:10.1371/journal.pone.0276135)
Supplement: Attachment — Submitted filename: Responses to the Reviewers 2.docx [file pone.0276135.s002.docx]

We appreciate all reviewers for reviewing our manuscript and giving useful comments to improve our manuscript again.
Additional Editor Comment:

Because of the unavailability of the original editor, I am handling your submission.
Your revised manuscript has been evaluated by three reviewers. Though two of them are more positive, Reviewer #2 raised serious concerns. In particular, as the reviewer pointed out, the results reported in 10.1073/pnas.1501372112 do not support the notion that CCR2+Ly6Chi cells contribute to pain. Accordingly, the mechanistic role of Ly6Chi monocytes in pain in your model needs to be further addressed, and your findings need to be better correlated with the literature.

Reviewers' comments:

Reviewer's Responses to Questions

Review Comments to the Author

Please use the space provided to explain your answers to the questions above. You may also include additional comments for the author, including concerns about dual publication, research ethics, or publication ethics. (Please upload your review as an attachment if it exceeds 20,000 characters)

Reviewer #1: The authors did an excellent work in addressing my comments and those raised by the different reviewers. The changes made by the authors greatly improve the manuscript. The remaining comments should be considered by the authors

Regarding the LTB4 plantar injection, a suggestion would be to evaluate cytokine concentration in plantar tissue to support the peripheral action of LTB4 on local cytokines.

Measurements of cytokine concentration in plantar tissue following LTB_4_ injection were performed previously. However, IL-1β, TNF- α and IL-6 elevation were small and not significantly different from vehicle injection group. (L298-300). The increase in LTB_4_ may not be directly related to local cytokine production.

The expression "mechanical hyperalgesia" is still present in the manuscript (abstract, description of the results, discussion) when the authors refer to von Frey's results. The authors have to change this expression since the up and down method with von Frey allows to assess tactile allodynia (or mechanical pain hypersensitivity as suggested in the first revision).

We checked again and replaced the term “Mechanical hyperalgesia” by “Mechanical pain hypersensitivity”.

Reviewer #2: This is the R1 version of the MS of Miho Asahara and colleagues.

Major points:

-In the answer: “Furthermore, according to the reference mentioned below (doi: 10.1186/s12990-015-0010-9), MPO-rich Ly6Chigh monocytes promote inflammation and tissue destruction via the release of proteases and inflammatory cytokines, such as TNF-α, in various inflammatory settings.” The cited reference does not show what was mentioned.

I apologize for citing the wrong reference. The correct reference is doi: 10.1084/jem.20070885.

-Previous question was: The discrepancy between the present finding and the previous finding (DOI:10.1073/pnas.1501372112) should be better explored with experiments. These papers are suggesting different cellular populations are involved in incisional pain. What are Ly6Chigh inflammatory monocytes doing in incisional pain and how LTB4 can affect their function? What is the explanation for the difference at day 1? At this time point there is no significant analgesia by BLT1 deficiency, but there is reduction of Ly6Chigh inflammatory monocytes and IL-1b and TNFa production. However, Ly6Chigh inflammatory monocytes are high at day 3 and we do not know the cytokine profile.

Answer was: Previous reports from other group (DOI: 10.1073/pnas.1501372112) have shown that monocytes (non-neutrophil myeloid cells) are more involved than neutrophils in postoperative pain. In our study, we showed that inhibition of monocytes by suppressing BLT1 signaling also contributes to pain control. BLT1-deficiency resulted in the suppression of monocyte infiltration on days 1 and day 3, and may also be involved in the suppression of cytokines on day 1.
Regarding the reason why the pain response in BLT1KO was not suppressed on day 1,
we speculate that inflammatory reaction was relatively strong on day 1; thus, the stronger monocyte suppression may be necessary for pain inhibition.
The data of cytokine production at day 3 was added to the new Fig 4.

Comment: Authors did not answer the question, but rather tried to blur what was questioned. The finding (DOI:10.1073/pnas.1501372112) disproved the contribution of inflammatory (CCR2(+)Ly6C(hi)) monocytes in post-incisional pain. The current Authors are working to demonstrate that Ly6Chigh inflammatory monocytes are contributing to post-incisional pain since BLT1 deficiency reduced their presence in the tissue and pain. In addition to this dismissed explanation of the Authors, Ly6Chigh inflammatory monocytes reduced when there was no reduction of pain, and again, Authors do not give a reasonable explanation, but rather speculate without data.

According to the FACS data of immune cells of planter tissue (DOI:10.1073/pnas.1501372112), Ly6C^high^ cell subset peaked on day1 and declined on day3. On the other hand, Ly6C^high^ cell subset increased on day1 and peaked on day3 in this study, suggesting that intensity of inflammation may be stronger than their model and the effect of Ly6C^high^ cell subset appeared more potently. We have described this in the text (L348-353).

-As mentioned by Reviewer #1, this MS needs more pain measurements. Significant reduction of allodynia was observed at days 2, 3 and 4. However, LTB4 production was significant only at 3h and day 1. LTB4 activates TRPV1, thus, there is need to evaluate thermal hyperalgesia.
-Another point that might be happening is that the Authors are not looking at the right cell type. Authors must assess CD11b(+)Ly6G(-) myeloid cells as previously demonstrated (DOI:10.1073/pnas.1501372112). This might be the reason why the results are not aligning.

Previous literature reported that CD11b^+^Ly6G^-^ myeloid cells are required for the mechanical hypersensitivity not for the thermal hypersensitivity that follows incisional wound-induced inflammation (DOI:10.1073/pnas.1501372112). In this study, we also focused on the role of inflammatory cells and LTB_4_-BLT1 signaling in the mechanical pain hypersensitivity following incision.

CD11b^+^Ly6G^-^ myeloid cells are assessed by analyzing the cell population after eliminating CD11b^+^Ly6G^+^ cells. Cell suspensions were first gated on forward-scatter (FSC-A) and side-scatter (SSC-A) to exclude debris. Neutrophils were then selected as double-positive for CD11b and Ly6G, quantified, and eliminated from further analysis. Non-neutrophil myeloid cells (CD11b^+^Ly6G^-^) were then gated based on Ly6C expression as Ly6C^high^ monocytes or Ly6C^low^ monocytes. Representative FACS plots of at 3day time point after incision from WT mice are shown in the below figures. Thus, CD11b^+^Ly6G^+^ myeloid cells (Neutrophil) and CD11b^+^Ly6G^-^Ly6C^+^ non-neutrophil myeloid cells (monocyte) were analyzed. In this study, FACS analysis of CD11b^+^Ly6G^+^ myeloid cells were shown in Figure 3 A and C. CD11b^+^Ly6G^+^ myeloid cells peaked on day1, and no significant difference was observed between BLT1WT and BLT1KO. On the other hand, CD11b^+^Ly6C^high^ subset were significantly reduced in BLT1KO mice than BLT1WT mice on day1 and day3 (Figure 3 B and D).

Ghasemlou et.al. showed that CD11b^+^Ly6G^+^ neutrophils do not contribute to the development of incisional pain by CD11b^+^Ly6G^+^ cell depletion study. Furthermore, depletion of CD11b^+^Ly6G^-^ cells were confirmed by CD11b-TK /GCV mice and these treatments showed significant increase of mechanical threshold. (DOI:10.1073/pnas.1501372112). Since most Ly6G^-^ cells (=Ly6C^+^) disappeared in this cell-depletion treatment, it was difficult to distinguish which subset group (Ly6Cl^ow^ or Ly6C^high^) was responsible for the incisional pain. They tried to see the effect of depletion of Ly6C^high^ and CCR2KO mice were used. In CCR2KO, reduction in all three subsets of Ly6C were shown; Ly6Cl^ow^ subset displayed a much smaller reduction at 24h than 3d. However, there was no significant difference in incisional pain responses between CCR2WT and CCR2KO.

Their results of the recruitment of CD11b^+^Ly6G^-^ cells contribute to incisional pain is aligning our results, but whether or not which subset of CD11b^+^Ly6C^+^ cells were responsible in incisional pain was remains to be elucidated. In this study, recruitment of the CD11b^+^Ly6C^high^ subset were reduced in BLT1KO and could be involved in the attenuation of pain response.

-Figure 4. Two cytokines known to induce pain (TNFa and IL-1b) have their production reduced by BLT1 deficiency at day 1 only, again, when there is no change in pain. I understand that some mediators cause nociceptor sensitization, however, this can be seen within some hours and not skipping one entire day. The time points of measurement after incision might also be influencing the results.

We consider that the reduction of the two cytokines reflects the suppression of monocyte infiltration by BLT1KO. However, whether a transient early increase of two cytokines is involved in the pain response cannot be resolved in this study. We speculate that the inflammatory reaction was relatively strong on day1, and, thus, stronger monocyte suppression may be necessary for pain inhibition during this period. Furthermore, in this study, neutrophil recruitment did not differ between KO and WT and peaked on day1, suggesting that other inflammatory mediators and chemokines released from neutrophils may sensitize peripheral nociceptors on day1. Description of peak time of neutrophil infiltration was corrected (L252).

-Authors also mention that they are presenting the individual results to allow verifying the variability but did not answer the question on whether results are normal and homogeneous.

Analysis of Normality were performed by Shapiro-Wilk test. Homogeneity of variance was confirmed by the Leven test using R statistical software version 4.2.1(R project for statistical computing). Description was added in the Method section. (L298-300)

Reviewer #3: The authors have responded appropriately to this reviewer’s comments.

However, the basal 50% paw withdrawal threshold shown in Figure 5 as an additional experiment to determine the effect of intraplantar injection of LTB4 (about 1.5 g) is quite different from that in Figure 1 (about 3.6-3.7 g). The authors should comment to this difference. Is the experimenter performing von Frey filament test who was blinded to the treatment and genotypes of mice one and the same person?

The mice used for behavior test in Figure 5 were 8 weeks old, and younger than the mice used in Figure 1(9-14 weeks old). It is possible that the age of the mice used affected the baseline threshold.

The experiments of assessment of pain behavior were performed in a blinded manner to the treatment and genotypes of mice.
